# High-Level Representations in Human Occipito-Temporal Cortex Are Indexed by Distal Connectivity

**DOI:** 10.1523/JNEUROSCI.2857-20.2021

**Published:** 2021-05-26

**Authors:** Jon Walbrin, Jorge Almeida

**Affiliations:** Proaction Laboratory, Faculty of Psychology and Educational Sciences, University of Coimbra, 3004-531 Coimbra, Portugal

**Keywords:** category-specificity, feature selection, functional connectivity, MVPA, object recognition, temporal cortex

## Abstract

Human object recognition is dependent on occipito-temporal cortex (OTC), but a complete understanding of the complex functional architecture of this area must account for how it is connected to the wider brain. Converging functional magnetic resonance imaging evidence shows that univariate responses to different categories of information (e.g., faces, bodies, and nonhuman objects) are strongly related to, and potentially shaped by, functional and structural connectivity to the wider brain. However, to date, there have been no systematic attempts to determine how distal connectivity and complex local high-level responses in occipito-temporal cortex (i.e., multivoxel response patterns) are related. Here, we show that distal functional connectivity is related to, and can reliably index, high-level representations for several visual categories (i.e., tools, faces, and places) within occipito-temporal cortex; that is, voxel sets that are strongly connected to distal brain areas show higher pattern discriminability than less well-connected sets do. We further show that in several cases, pattern discriminability is higher in sets of well-connected voxels than sets defined by local activation (e.g., strong amplitude responses to faces in fusiform face area). Together, these findings demonstrate the important relationship between the complex functional organization of occipito-temporal cortex and wider brain connectivity.

**SIGNIFICANCE STATEMENT** Human object recognition relies strongly on OTC, yet responses in this broad area are often considered in relative isolation to the rest of the brain. We employ a novel connectivity-guided voxel selection approach with functional magnetic resonance imaging data to show higher sensitivity to information (i.e., higher multivoxel pattern discriminability) in voxel sets that share strong connectivity to distal brain areas, relative to (1) voxel sets that are less strongly connected, and in several cases, (2) voxel sets that are defined by strong local response amplitude. These findings underscore the importance of distal contributions to local processing in OTC.

## Introduction

Human object recognition is a rapid process that relies heavily on occipito-temporal cortex (OTC; Grill-Spector and Malach, 2004), and there have been extensive efforts to fully characterize the complex functional organization of this area (Grill-Spector and Weiner, 2014; Peelen and Downing, 2017; Op de Beeck et al., 2019). Convergent functional magnetic resonance imaging (fMRI) findings show coarse-grain organization of OTC as evidenced by spatially clustered category-preferring responses, that is, regions that show enhanced fMRI response amplitude for one category over others (e.g., faces, tools, and places/scenes; Kanwisher et al., 1997; Epstein and Kanwisher, 1998; Chao and Martin, 2000; Downing et al., 2006; Beauchamp and Martin, 2007; Almeida et al., 2013; Kristensen et al., 2016), along with finer-grain organization via patchy organization of the OTC (i.e., sparsely distributed cortical patches that respond strongly to different information; Grill-Spector et al., 2006; Weiner and Grill-Spector, 2010) that are well captured with multivoxel pattern analysis (MVPA) techniques (Haxby et al., 2001; Kamitani and Tong, 2005).

However, a complete understanding of the functional architecture of OTC must account for how this broad area interfaces with the wider brain. Indeed, connectivity is a major constraint on the functional organization of cerebral cortex in general, such that the functional response of a given region is partially determined by the integration of relevant information shared via structural and functional connectivity to other brain regions (e.g., Garcea et al., 2019; Lee et al., 2019; Mahon and Caramazza, 2011; Sporns and Zwi, 2004; Sporns, 2014). More specifically, category-preferring OTC responses are functionally coupled with, and modulated by, distal regions that share the same category preference (e.g., tool responses in medial fusiform gyrus (MFus) are shaped by inferior parietal cortex; Amaral et al., 2021; Chen et al., 2017; Garcea et al., 2019; Lee et al., 2019); similarly, OTC responses for multiple visual categories (e.g., faces, objects, bodies, and places) can be reliably predicted from patterns of white matter connectivity to the wider brain (Saygin et al., 2012, 2016; Osher et al., 2016).

The preceding evidence demonstrates a clear relationship between distal connectivity and functional local OTC responses at the level of individual voxels. However, the extent to which connectivity relates to complex distributed functional responses (i.e., multivoxel pattern decoding) is not yet understood. Here, we show that the discriminability of distributed multivoxel response patterns in OTC is related to, and importantly, can be indexed by, patterns of distal connectivity; that is, sets of voxels that afford high pattern discriminability of different object categories can be identified by the strength of connectivity that they share with distal brain areas. Specifically, our results demonstrate that (1) most-connected gray matter voxel sets consistently yield higher pattern discriminability than least-connected sets do, and (2) most-connected voxel sets are partially distinct from, and in several cases, afford significantly higher pattern discriminability than most-activated voxel sets do (i.e., sets defined by strongest amplitude responses). In summary, these findings demonstrate a compelling relationship between distal connectivity and locally distributed functional responses in OTC.

## Materials and Methods

### 

#### 

##### Participants

Twenty right-handed undergraduate adult participants (mean age 22.1 years; SD, 5.4; 14 females) gave informed consent and were reimbursed with university course credit. We did not perform a power analysis to determine the number of participants to be tested in this study. Instead, we defined the number of participants by following previously published reports that described group-level object category MVPA discriminations (and even within-category discriminations) with a similar or smaller number of participants (Op de Beeck et al., 2008b; Weiner and Grill-Spector, 2010; Hafri et al., 2017; Lee et al., 2019). Head motion was not excessive for any subject (i.e., no >2 mm scan-to-scan spikes), so all data were used. Ethical procedures were approved by the Faculty of Psychology and Educational Sciences of the University of Coimbra ethics board.

##### Experimental design and statistical analyses

A repeated-measures design was used to assess decoding performance across the following three factors: voxel selection type (e.g., most-connected, least-connected voxel set), target region [e.g., tool-preferring medial fusiform gyrus and posterior middle temporal gyrus (PMTG)], binary decoding comparison (e.g., tools vs faces, tools vs places). Three-way repeated-measures ANOVAs were used for all decoding analyses, with several noted exceptions (see below, Matched activation analyses). For conciseness, only ANOVA terms involving voxel selection type are reported here (Extended Data Figures 1-1–2.2); specifically, we only report these effects at the highest descriptive level (i.e., for significant interactions involving voxel selection type, we report the corresponding *post hoc* test; in the absence of a significant interaction term, we report the main effect of voxel selection type). A Bonferroni-corrected threshold was calculated for each set of *post hoc t* tests (two tailed) involving voxel selection type, and all reported tests survive correction unless otherwise stated.

##### MRI scanning parameters

Scanning was performed with a Siemens MAGNETOM Trio, A Tim System 3T MRI Scanner (Siemens Healthineers) with a 12-channel head coil at the University of Coimbra. Functional images were acquired with the following parameters: T2*-weighted single-shot echo-planar imaging pulse sequence, repetition time (TR) = 2000 ms, echo time (TE) = 30 ms, flip angle = 90°, 40 interleaved axial slices (no gap), acquisition matrix = 96 × 96 with field of view = 256 mm, with a voxel size of 2.3 × 2.3 × 3 mm. Structural T1-weighted images were obtained using a magnetization prepared rapid gradient echo (MPRAGE) sequence with the following parameters: TR = 2530 ms, TE = 3.29 ms, in 1.7 ms steps, total acquisition time = 136 s, FA = 8°, acquisition matrix = 256 × 256, with field of view 256 mm, and voxel size = 1 mm^3^.

##### Task

Participants completed six runs of a blocked-design task, where they centrally fixated gray-scaled images (400 × 400 pixels; ∼10° of visual angle) of tools, faces, and places (animal images as well as phase-scrambled variants of these categories were also presented but were not analyzed here.). Each run consisted of alternating 6 s blocks of stimuli and 6 s fixation, with 16 s fixation at the beginning and end of each run (run length: 176 s = 88 TRs); two blocks were presented for each of the categories (and one block for each of the phase-scrambled conditions). Block order was randomized across runs.

##### Preprocessing

Preprocessing was performed with SPM12. This entailed slice-timing correction, realignment (and reslicing), coregistration, and segmentation. Segmented gray matter maps were coregistered and warped to subject's functional image space for later masking out white matter voxels. A duplicate set of functional data were normalized and smoothed for the sole purpose of identifying group-level activation peaks for creating a search space for each target area. All default SPM12 parameters were used, except for normalized data where output voxel size was 3 mm^3^ and a 6 mm^3^ full width at half maximum (FWHM) Gaussian smoothing kernel was used.

General linear model estimation was performed in SPM12, and all analyses were performed in subject space. Block durations and onsets for each experimental condition were modeled by convolving the corresponding box-car time course with a canonical hemodynamic response function (without time or dispersion derivatives), with a high-pass filter of 256 s and autoregressive AR(1) model. Beta maps were generated on a run-wise basis, yielding one regressor per condition, along with six rigid-motion regressors (and an intercept regressor). T-maps were estimated for the contrasts described below.

The preprocessed functional data were duplicated, and denoising was performed with the CONN toolbox (Whitfield-Gabrieli and Nieto-Castanon, 2012) by regressing out task-related effects (i.e., hemodynamic response convolved with blocks for each condition), along with other head motion (6 rigid-motion regressors + 6 first-order temporal derivatives) and physiological noise-related variables (mean global signal estimated from all white matter and cerebrospinal fluid voxels, along with outlier scan removal), and bandpass filtered (0.01–0.1 Hz). Previous work shows that this approach successfully removes task-related signal, resulting in time course data that is very similar to resting-state fMRI signal (e.g., Fair et al., 2007).

##### Voxel selection

We used a connectivity-guided voxel selection approach in six target regions: tool-preferring MFus and PMTG, face-preferring fusiform face area (FFA) and occipital face area (OFA), and place-preferring parahippocampal place area (PPA) and occipital place area (OPA). The following regions outside OTC were also used for connectivity seeding: tool-preferring inferior parietal lobe (IPL) and superior parietal lobe (SPL), face-preferring superior temporal sulcus (STS-F), and place-preferring retrosplenial cortex (RSC). Thus, voxel selection within a given target region depended on connectivity to all other regions (both within and outside of OTC) that shared the same category preference (e.g., PMTG, IPL, and SPL served as seed regions for voxel selection in MFus.). Analyses were restricted to left-hemisphere tool regions, and right-hemisphere face and place regions, based on widely observed hemispheric asymmetries (Downing et al., 2006); however, we also observed the same pattern of results in the opposite hemisphere for each set of regions.

Target region masks (i.e., search spaces for voxel selection) were created by centering a 15 mm radius sphere at the most-activated voxel (uncorrected *p* < 0.05), based on group-level activation in normalized space. For tool-, face-, and place-preferring regions, activation was based on the following *t*-contrasts: tools > [faces + places + animals], faces > [tools + places + animals], and places > [tools + faces + animals], respectively. Voxels that overlapped between two adjacent search spaces for the same category (e.g., OFA and FFA) were removed; search spaces between categories (e.g., tool-preferring MFus and face-preferring FFA) were free to overlap. Target region masks were then inverse registered to each subject's own brain space, and white-matter (and cerebellum) voxels were removed. All target region masks contained >300 gray-matter voxels. Seed regions were then defined as the 100 most-activated voxels (for the same contrasts described above, based on each subject's own activity, i.e., *t* values) within each target region.

For each target region, a functional connectivity matrix was calculated that described the time course correlation (Fisher-transformed Pearson's *r* coefficient) between each voxel and all other same-category seed voxels (e.g., 350 MFus target regions voxels × 100 PMTG + 100 IPL + 100 SPL seed region voxels). The mean correlation for each target region voxel (across all seed regions) was then obtained, and the 100 most highly connected and 100 least highly connected target region voxels were selected. We inspected the average group-level connectivity values for each voxel set (e.g., average connectivity to seed regions across most-connected voxels in MFus) by extracting subjects' median voxel set values (i.e., Fisher transformed Pearson's *r* coefficient) and mean averaging them to create a group-level estimate of connectivity per set. Most-connected sets showed positive connectivity to seed regions (group-averaged Fisher transformed Pearson's *r* values ranging from 0.052 to 0.067 across the 6 target regions), whereas least-connected sets showed weaker negative connectivity to seed regions (ranging from −0.043 to −0.024 across the 6 target regions).

We also compared sets of most-connected voxels with sets of 100 most highly activated voxels based on the corresponding *t*-contrast for each decoding analysis (e.g., tools > faces *t* values were used for tools versus faces decoding). Importantly, because of potential circularity problems (Kriegeskorte et al., 2009; e.g., exaggerated tools versus faces decoding accuracy might result if voxel sets are defined with the exact same data), data were independently split for voxel selection and decoding as follows. Subject data (both task and task-regressed connectivity datasets) were divided into three splits (two runs each). A leave-one-split-out approach was adopted for generating and testing both connectivity and activity voxel sets, where one split of data was used for voxel selection and the remaining two splits were used in the corresponding decoding fold (iterated three times, so that each split was used for voxel selection). Within each target region, voxels did not overlap for the most-connected and least-connected sets, but overlap was unconstrained between most-connected and most-activated voxel sets.

##### Signal-to-noise-ratio analysis

To test whether subtle differences in signal-to-noise-ratio (SNR) might explain a potential decoding advantage in most-connected relative to least-connected voxel sets, that is, higher SNR in most-connected voxel sets might partially account for higher decoding compared with least-connected voxel sets, we directly compared SNR between voxel sets as follows.

Whole-brain maps that describe the voxel-wise temporal SNR (i.e., mean signal amplitude/SD; Triantafyllou et al., 2005) for each run of task data were generated for each subject. Mean SNR values for most-connected and least-connected voxel sets were then obtained for each subject across all six runs of data (within each target area, and averaged across voxel sets from all 3 data splits), and entered into two-way ANOVA (voxel selection type × region). These analyses revealed an effect in the opposite direction; that is, SNR was slightly higher in least-connected than most-connected voxel sets (main effect of voxel selection, *F*_(1,19_) = 25.51, *p* < 0.001, η_p_^2^ = 0.573; most-connected > least connected, *post hoc* contrast: *t*_(19)_ = −5.05, *p* < 0.001), indicating that any potential decoding advantage in most-connected voxel sets relative to least-connected voxel sets is not attributable to higher SNR in most-connected voxel sets.

##### Multivoxel pattern decoding

Decoding was implemented with the CoSMoMVPA Toolbox (Oosterhof et al., 2016). A split-half Pearson's *r* correlation decoding approach was used (Haxby et al., 2001) as a measure of discriminability between each relevant pair of conditions. This is a powerful decoding approach that performs equivalently to commonly used linear classifiers (Misaki et al., 2010).

Decoding was performed across three decoding folds (i.e., voxels selected with 1 data split and decoding performed with the 2 left-out data splits, with a different data split used for voxel selection for each decoding fold). Two decoding comparisons were run for each category to ensure the generalizability of effects (i.e., tool-preferring regions: tools vs faces and tools vs places; face-preferring regions: faces vs tools and faces vs places; place-preferring regions: places vs tools and places vs faces). For each decoding comparison pair (e.g., tools vs faces), patterns for each condition were correlated across the two designated decoding splits of data (i.e., two runs per split), yielding a 2 (split) × 2 (category) confusion matrix, where the mean between-category correlation (off-diagonal cells) was subtracted from the mean within-category correlation (on-diagonal cells); thus, a positive decoding accuracy denotes greater within-category than between-category decoding [e.g., (tools-to-tools correlation + faces-to-faces correlation] > tools-to-faces correlations, across splits]. Subjects' decoding accuracy values were mean averaged across decoding folds and entered into three-way repeated-measures ANOVAs (i.e., voxel selection type × region × decoding comparison), for each set of category-preferring regions, separately.

##### Matched-activation analyses

To ensure that any differences between most-connected and least-connected voxel sets were not confounded by local activation to category information (e.g., differences between the 2 voxel sets in FFA might result from differences in mean activation to faces), a series of matched-activation analyses was performed. This entailed selecting strongly connected and weakly connected voxel sets under the constraint that they did not statistically differ by their average activation (For face regions, *t* values were matched for each corresponding decoding analysis, e.g., faces > tools *t* values were used for faces vs tools decoding.). This was achieved with a permutation approach as follows.

Voxels in each target region were median split by their mean connectivity values (i.e., mean connectivity correlation value to all seed voxels). Two random subsets of 100 voxels, one each from the highest and lowest half-splits, were then drawn and compared to ensure that their average activation values—voxel *t* values—did not differ when compared via an independent *t* test. Ten thousand subset comparisons were performed but, crucially, only subset pairs with nonsignificant independent *t* test statistics were retained. Decoding was then performed with these voxel sets and averaged to create stable decoding estimates for the strongly connected and weakly connected voxel sets, respectively.

This analysis was repeated across three statistical thresholds, retaining pairs of voxel sets that did not differ: (1) at a liberal threshold (i.e., two-tailed independent *t* test *p* values > 0.10), (2) at an intermediate threshold (i.e., independent *t* test statistics within the range of *t* = +0.5 to −0.5), and (3) at a strict threshold, where voxel set pairs were only accepted when the average activation was lower in strongly connected sets (i.e., independent *t* test statistics within the negative range of *t* = 0 to −0.5).

We initially ran three-way repeated-measures ANOVAs (voxel selection type × region × decoding comparison) for tools, faces, and places separately as in the main analyses to test these results. However, we observed reduced degrees of freedom for these analyses, indicating that these constraints were not always met in all subjects and regions (e.g., connectivity and activity were less independent of each other for some regions and in some subjects so that mean activation always differed between voxel sets). To preserve statistical power, we ran follow-up two-way repeated-measures ANOVAs (voxel selection type × decoding comparison) for each region separately if the initial three-way ANOVA indicated that at least three subjects did not meet this constraint; if the constraint was met in one region but not the other in a given subject, this would allow for those data to be retained when testing the surviving region. Specifically, three-way ANOVA results are reported for face regions as only one subject failed to meet this constraint across all three matched-activation analysis thresholds. Because of higher subject dropout for the other three-way ANOVAs (i.e., for tools and places), region-wise two-way ANOVA results are reported for the two more conservative thresholds, but three-way ANOVA results are reported at the most liberal threshold, where only two subjects failed to meet this constraint. The number of remaining subjects per analysis is reported below in Results.

##### Good seed searchlight analysis

To complement the main analyses that use *a priori* seed regions, we ran a searchlight analysis to determine which regions across the entire brain constituted good seeds (i.e., regions with connectivity that yields higher decoding in most-connected compared with least-connected target region voxels). For each target region (e.g., MFus), a searchlight consisting of ∼100 contiguous voxels was centered on each given gray-matter voxel of the brain (excluding the given target region), and the mean time course for those voxels was correlated with the target region for voxel selection. Decoding was performed, and accuracy values were then assigned to the central voxel of the corresponding searchlight. This was performed for each subject across all analysis variants (i.e., for each category, 2 target regions × 2 binary decoding comparisons × 2 voxel sets; i.e., most connected and least connected).

The same decoding approach as in the main analyses (i.e., with *a priori* seeds) was adopted here, except that decoding was performed with all six runs of data in a single decoding fold (i.e., where run-averaged patterns between the 3 odd and 3 even runs were correlated), rather than adhering to the data split scheme imposed in the previous analyses (i.e., 3 decoding folds). This was done for the following reasons: (1) because activation was not used for comparative voxel selection here, data circularity problems do not apply, and (2) this demonstrates the generalizability of the distal connectivity decoding effect with a different decoding scheme (We also ran these analyses with the same split scheme as in the main analyses and observed virtually identical results.).

For group-level inference, paired *t* tests with threshold-free cluster enhancement (Smith and Nichols, 2009) based on 10,000 Monte Carlo simulations were run with subjects' most-connected and least-connected voxel selection searchlight maps (Input maps were normalized and smoothed with a 6 mm FWHM kernel.). The resulting group-level maps were thresholded at *Z* > 1.65 and projected to a surface rendered brain in SPM12 for visualization. In short, these maps show regions that constitute good seeds, yielding a significant decoding effect (i.e., seeding from these regions results in higher decoding for the most-connected than least-connected voxel sets in the corresponding target region).

##### Data availability

The data and accompanying code are available on request from the authors.

## Results

**Figure 1.**
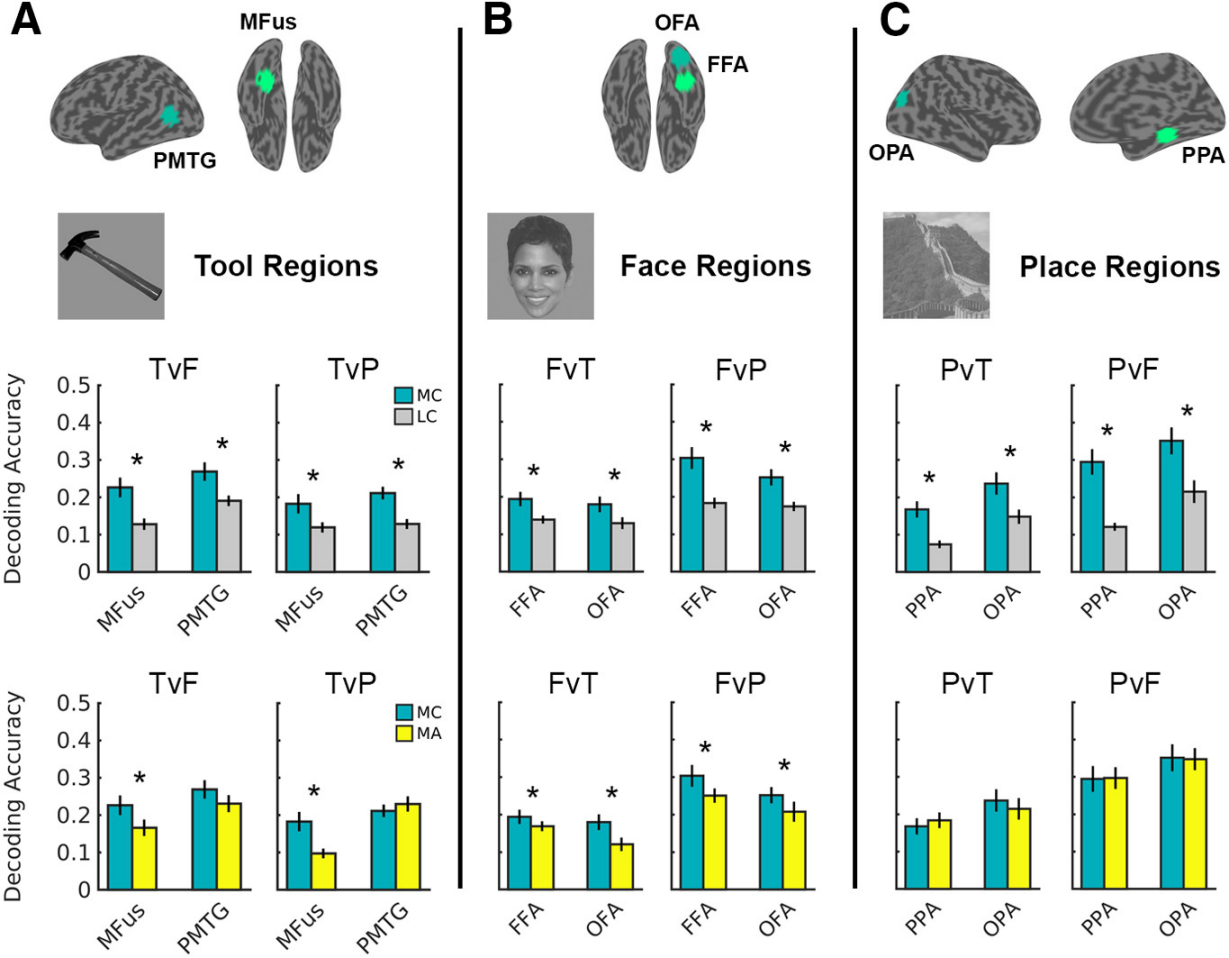
***A–C***, Mean decoding accuracy for most-connected (MC), least-connected (LC), and most-activated (MA) voxel sets, for tool (***A***), face (***B***), and place (***C***) regions. Upper row bar charts: MC versus LC decoding. Lower row bar charts: MC versus MA decoding. Tool regions: MFus, PMTG. Face regions: FFA, OFA. Place regions: PPA, OPA. Decoding comparisons: TvF, Tools versus faces; TvP, tools versus places; FvT, faces versus tools; FvP, faces versus places; PvT, places versus tools; PvF, places versus faces. * = significant MC > LC effect (Bonferroni corrected, *p* < .05). Error bars are SEM. Target region search spaces are shown on a surface brain, along with example stimuli in the upper portion of the figure. For full statistics, see Extended Data Figures 1-1 and 1-2.

Across all six target regions, higher decoding accuracy was observed for most-connected than least-connected voxel sets (Fig. 1*A*–*C*, upper row bars; Extended Data Fig. 1-1). This effect was shown for both tool regions (i.e., MFus and PMTG; main effect of voxel selection type: *F*_(1,19)_ = 46.85, *p* < 0.001, η_p_^2^ = 0.711), both face regions [i.e., FFA and OFA; voxel selection type × decoding comparison (interaction): *F*_(1,19)_ = 14.64, *p* < 0.001, η_p_^2^ = 0.435; faces versus places (*post hoc*): *t*_(24.30)_ = 5.79, *p* < 0.001; faces versus tools (*post hoc*): *t*_(24.30)_ = 3.09, *p* = 0.005], and both place regions [i.e., PPA and OPA; voxel selection type × decoding comparison (interaction): *F*_(1,19)_ = 12.11, *p* = 0.003, η_p_^2^ = 0.389; places versus faces (*post hoc*): *t*_(28.32)_ = 7.69, *p* < 0.001; places versus tools (*post hoc*): *t*_(28.32)_ = 4.52, *p* < 0.001]. Thus, decoding accuracy is consistently higher in voxel sets that are the most- rather than least-distally connected.

10.1523/JNEUROSCI.2857-20.2021.f1-1 Figure 1-1 Note: Three-way ANOVAs when comparing most-connected and least-connected voxel sets. Significant effects are indicated in bold; *post hoc* tests (following significant interactions involving the factor voxel selection are shown in gray cells. Download Figure 1-1, DOCX file.

**Figure 2.**
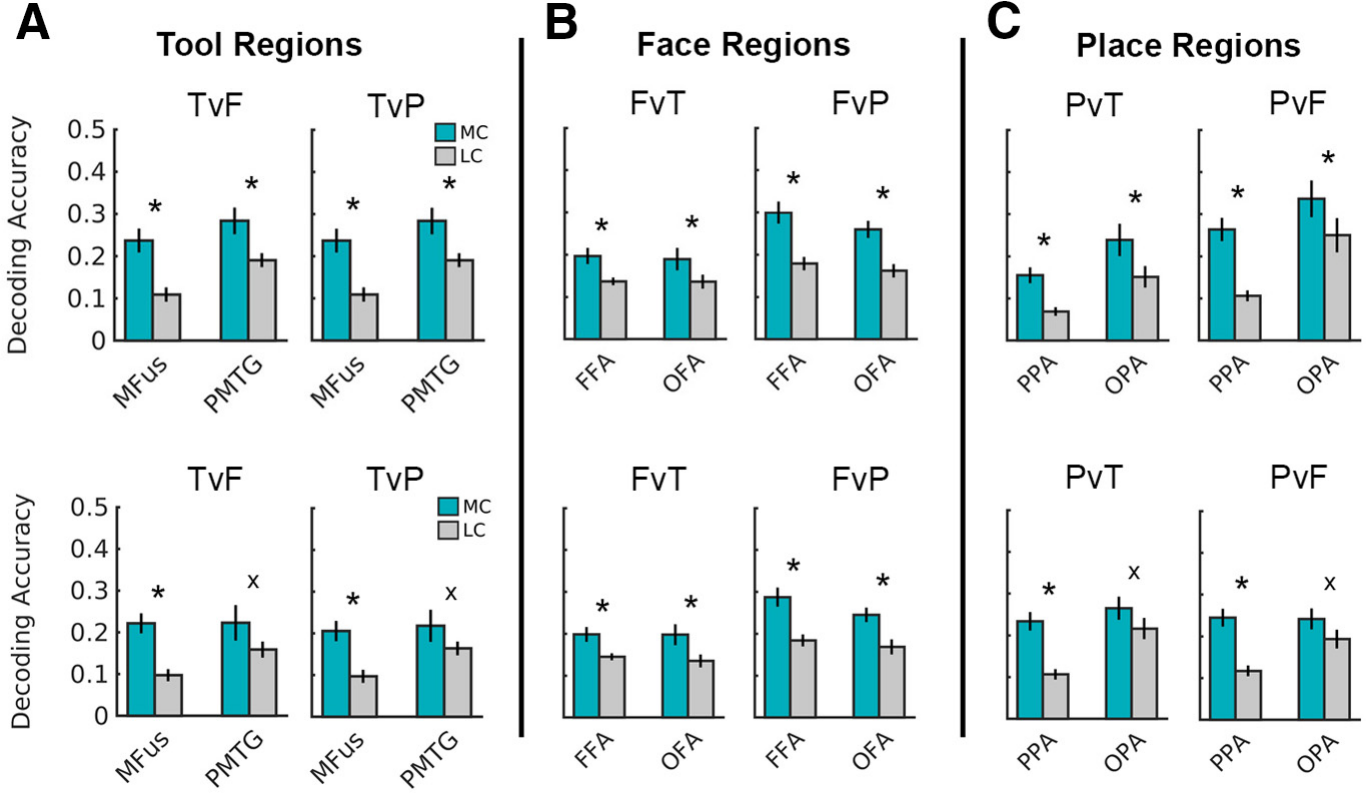
***A–B***, Mean decoding accuracy for most-connected (MC) and least-connected (LC) voxel sets for matched activation analyses for tool (***A***), face (***B***), and place (***C***) regions. Upper row bars: liberal nondifferent activation threshold (*p* > 0.10). Lower row bars: strictest nondifferent activation threshold (negative *t* values between 0 to −0.5). Tool regions: MFus, PMTG. Face regions: FFA, OFA. Place regions: PPA, OPA. Decoding comparisons: TvF, tools versus faces; TvP, tools versus places; FvT, faces versus tools; FvP, faces versus places; PvT, places versus tools; PvF, places versus faces. * = significant effect (Bonferroni corrected, *p* < .05). x = Trend nonsignificant/underpowered analyses (PMTG, *N* = 8; OPA, *N* = 13). Error bars are SEM. For full statistics, see Extended Data Figures 2-1 and 2-2.

10.1523/JNEUROSCI.2857-20.2021.f1-2 Figure 1-2 Note: Three-way ANOVAs when comparing most-connected and most-activated voxel sets. Significant effects are indicated in bold; *post hoc* tests (following significant interactions involving the factor voxel selection) are shown in gray cells. Download Figure 1-2, DOCX file.

Previous evidence shows that distal functional connectivity is correlated with task-based activation in OTC (Chen et al., 2017; Amaral et al., 2021). It is therefore possible that most-connected voxels are effectively the same as those showing strongest local activation, and therefore most-connected and most-activated voxel sets might yield equivalent decoding performance. We tested this by comparing decoding accuracy in most-connected voxel sets with those that were most-activated by their preferred stimulus category (both sets were free to overlap). Interestingly, decoding accuracy was never statistically lower in most-connected relative to most-activated voxel sets; indeed, decoding in most-connected voxel sets was almost always equal or higher than decoding in most-activated voxel sets (Fig. 1*A–C*, lower row bars; Extended Data Fig. 1-2). For tool-preferring regions, decoding accuracy was significantly higher for most-connected relative to most-activated voxel sets (voxel selection × decoding comparison × region interaction: *F*_(1,19)_ = 5.04, *p* = 0.037, η_p_^2^ = 0.210) in MFus [tools vs faces (*post hoc*): *t*_(74.31)_ = 3.16, *p* = 0.002; tools vs places (*post hoc*): *t*_(74.31)_ = 4.45, *p* < 0.001], but this trend was not significant in PMTG [tools vs faces (*post hoc*): *t*_(74.31)_ = 1.99, *p* = 0.050; tools vs places (*post hoc*): *t*_(74.31)_ = −0.97, *p* = 0.335]. By contrast, this effect was significant in both FFA and OFA (main effect of voxel selection type: *F*_(1,19)_ = 10.62, *p* = 0.004, η_p_^2^ = 0.358), but was not significant in either place region (all voxel selection ANOVA terms: *p* > 0.115).

These results show that decoding performance is not equivalent in most-connected and most-activated voxel sets in several areas. Nevertheless, we further sought to test the relative independence of the effects observed in the original analysis by looking at whether the decoding differences between most-connected and least-connected voxel sets remain when potential differences in average activation between sets are controlled; that is, does greater decoding in most-connected- than least-connected voxel sets remain when average activation between the two sets is closely controlled?

To test this, we ran a matched-activation permutation analysis where we median split each target area by voxel connectivity values and randomly drew subsets of 100 strongly connected and 100 weakly connected voxels but, crucially, only compared decoding performance in sets that did not statistically differ by their average activation (i.e., voxel *t* values; see above, Materials and Methods). In the first variant of this analysis, we retained pairs of voxel sets that did not statistically differ at a relatively liberal threshold (i.e., two-tailed *p* > 0.10) when running an independent *t* test between the two voxel sets' activation values (voxel *t* values).

As before, decoding accuracy was significantly higher for most-connected than least-connected voxel sets (Fig. 2*A*–*C*, upper row bars; Extended Data Fig. 2-1) in both tool regions (main effect of voxel selection: *F*_(1,17)_ = 52.04, *p* < 0.001, η_p_^2^ = 0.754); both face regions [voxel selection type × decoding comparison (interaction): *F*_(1,19)_ = 23.22, *p* < 0.001, η_p_^2^ = 0.550; faces versus places (*post hoc*): *t*_(23.57)_ = 6.60, *p* < 0.001; faces versus tools (*post hoc*): *t*_(23.57)_ = 3.43, *p* = 0.002]; both place regions [voxel selection type × decoding comparison (interaction): *F*_(1,17)_ = 10.39, *p* = 0.005, η_p_^2^ = 0.379; places versus faces, (*post hoc*): *t*_(25.9)_ = 7.81, *p* < 0.001; places versus tools (*post hoc*): *t*_(25.9)_ = 4.79, *p* < 0.001].

10.1523/JNEUROSCI.2857-20.2021.f2-1 Figure 2-1 Note: Three-way ANOVAs when comparing most-connected and most-activated voxel sets (matched activation; *p* > .10). Significant effects are indicated in bold; *post hoc* tests (following significant interactions involving the factor voxel selection) are shown in gray cells. Download Figure 2-1, DOCX file.

10.1523/JNEUROSCI.2857-20.2021.f2-2 Figure 2-2 Note: Three-way and two-way ANOVAs when comparing most-connected and most-activated voxel sets (matched activation *t* 0 to −0.5). Significant effects are indicated in bold; *post hoc* tests (following significant interactions involving the factor voxel selection) are shown in gray cells. Download Figure 2-2, DOCX file.

We next repeated this analysis under two stricter thresholds by only retaining voxel set pairs where (1) average activation was more closely matched between the two sets (i.e., independent *t* tests that yielded *t* statistics within the range of +0.5 to −0.5), and (2) average activation was lower in most-connected voxel sets (i.e., independent *t* tests that yielded negative *t* statistics with the range of 0 to −0.5). Given these conservative criteria, we anticipated that these constraints would not be met in all subjects, and therefore the number of surviving subjects is reported for each analysis.

Under the intermediate threshold (i.e., *t* statistics between +0.5 to −0.5), higher decoding accuracy was observed for most-connected than least-connected voxel sets in all regions. For MFus, FFA, OFA, and, PPA, 19 of 20 subjects met this constraint (MFus: *F*_(1,18)_ = 19.30, *p* < 0.001, η_p_^2^ = 0.517; FFA and OFA: *F*_(1,18)_ = 23.59, *p* < 0.001, η_p_^2^ = 0.567; PPA: *F*_(1,18)_ = 43.46, *p* < 0.001, η_p_^2^ = 0.707). These effects were also significant in PMTG and OPA, where 13 and 14 subjects remained, respectively (PMTG: *F*_(1,12)_ = 6.97, *p* = 0.022, η_p_^2^ = 0.367; OPA: *F*_(1,13)_ = 4.83, *p* = 0.047, η_p_^2^ = 0.271).

Under the strictest constraint (i.e., *t* statistics between 0 to −0.5), higher decoding accuracy was (again) observed for most-connected than least-connected voxel sets across all regions (Fig. 2*A*–*C*, lower row bars; Extended Data Fig. 2-2). This trend was statistically significant in all regions where this constraint was met for at least 17 of 20 subjects: MFus, FFA, OFA, and PPA [MFus F (1,18) = 18.66, *p* < 0.001, η_p_^2^ = 0.509; FFA and OFA (*post hoc* test; faces vs tools): *t*(24.97) = 3.26, *p* = 0.003; FFA and OFA (*post hoc* test; faces vs places): *t*_(24.97)_ = 5.04, *p* < 0.001; PPA: *F*_(1,16)_ = 35.00, *p* < 0.001, η_p_^2^ = 0.686]. In PMTG and OPA, these analyses were underpowered (i.e., only 8 and 13 subjects remained, respectively) and did not reach significance (PMTG: *F*_(1,7)_ = 1.51, *p* = 0.258, η_p_^2^ = 0.178; OPA: *F*_(1,12)_ = 2.81, *p* = 0.120, η_p_^2^= 0.190). Although these trends are evident in Figure 2*A–C* (lower row), these results show a lesser degree of independence between connectivity and activity measures in PMTG and OPA than the other regions.

Together, these analyses show strong decoding performance in highly connected voxel sets; importantly, these distally well-connected voxel sets demonstrate a degree of independence from—and therefore, are not merely confounded by—local voxel activation (*t* values).

**Figure 3.**
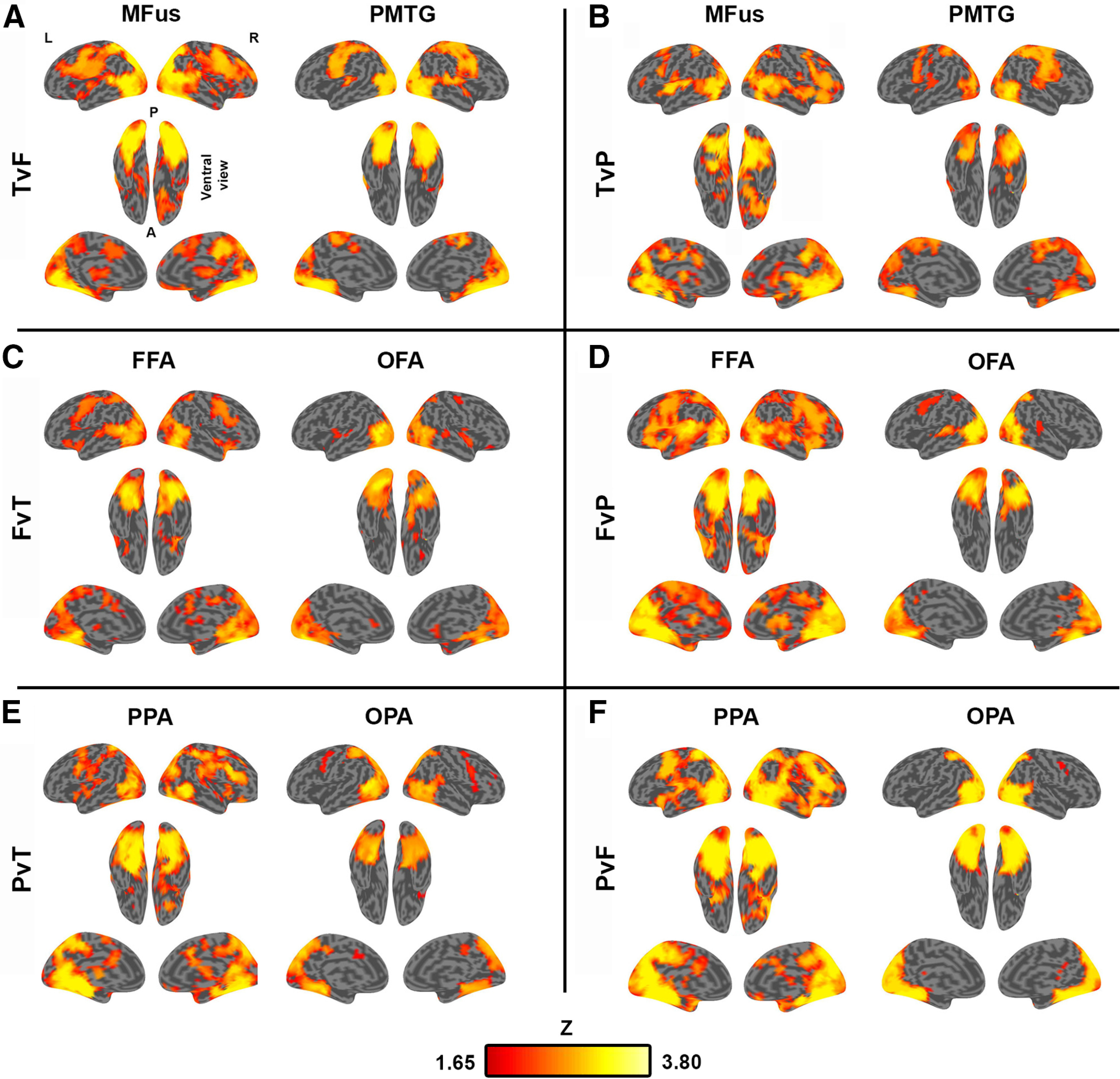
Group searchlight maps showing good seed areas for each target region. *z*-score voxel intensities (threshold-free cluster enhancement paired *t* test; *Z* threshold > 1.65) show regions that seed significantly higher decoding accuracy for most-connected than least-connected voxels within a given target region. ***A–F***, Decoding comparisons: ***A***, tools versus faces (TvF); ***B***, tools versus places (TvP); ***C***, faces versus tools (FvT); ***D***, faces versus places (FvP); ***E***, places versus tools (PvT); ***F***, places versus faces (PvF). Tool regions: MFus, PMTG. Face regions: FFA, OFA. Place regions: PPA, OPA.

Finally, we implemented whole-brain searchlight analyses (Fig. 3) for each target region; these analyses revealed regions (beyond *a priori* seed regions used in the preceding analyses) that afford good seeding (i.e., regions with distal connectivity that yields higher decoding in most-connected rather than least-connected voxel sets). Diffuse patterns of strong seeding in the wider brain were shown for all target regions. Notably, good seeding was observed in bilateral posterior temporal cortex (coincident with category-preferring OTC regions) and early visual cortex, as well as dorsal attention and task-general cognitive control regions (e.g., anterior inferior parietal sulcus, frontal eye fields, and precentral gyrus), and this coverage is comparable to previously observed functional connectivity patterns between OTC and the wider brain (Vogel et al., 2012; Hutchison et al., 2014).

## Discussion

Here, we emphasize two main findings. First, complex functional responses in OTC are strongly related to patterns of connectivity to distal brain areas (i.e., gray-matter voxel sets that share strong functional connectivity with the wider brain yield consistently better pattern discriminability than lesser-connected sets do, across all tested information categories). These findings align with previous demonstrations that local OTC responses are shaped by distal connectivity with the wider brain (Saygin et al., 2012, 2016; Osher et al., 2016; Chen et al., 2017; Garcea et al., 2019; Lee et al., 2019; Amaral et al., 2021); and the more general proposal that functional brain responses are strongly determined by the integration of relevant information shared via structural and functional connectivity to the wider brain (Varela et al., 2001; Sporns, 2014; Sporns and Zwi, 2004; Mahon and Caramazza, 2011; Saygin et al., 2012, 2016; Park and Friston, 2013; Osher et al., 2016; Ruttorf et al., 2019). Ultimately, local computations and the organization of representational content in OTC are dependent on interactions between connectivity-constrained neural assemblies that are likely dedicated to achieving particular computational goals (e.g., coordinated tool use, or face-to-face social interaction; Op de Beeck et al., 2008a; Mahon, 2015; Peelen and Downing, 2017).

Second, most-connected voxels are not merely those that are most activated, as shown by higher pattern discriminability for most-connected relative to most-activated voxel sets in several regions (i.e., MFus, FFA, and OFA, and performed equivalently in all other regions), and further, the decoding advantage for most-connected than least-connected voxel sets remains when average activation (voxel *t* values) of the two sets is constrained. These results are consistent with the observation that even voxels with weak amplitude responses may contribute meaningfully to pattern discrimination (Haxby et al., 2001; Kamitani and Tong, 2005; Weiner and Grill-Spector, 2010); as such, the informativeness of weakly activated voxels may be captured via connectivity to the wider brain.

The decoding differences shown here between most-connected and most-activated voxel sets might, at first glance, seem to conflict with previous evidence that emphasizes a statistical similarity between connectivity and activity measures; for example, category-specific activation in fusiform gyrus is correlated with the degree of functional connectivity to seed areas that share the same category preference (e.g., voxel-level activation to tool stimuli correlates with the voxel-level connectivity to tool-preferring IPL; Chen et al., 2017). Similarly, although matched-activation analyses shown here demonstrate a decoding advantage in most-connected relative to least-connected voxel sets when controlling for average activation between the two sets, these analyses also show that connectivity and activity are certainly related (i.e., activation could not be matched between sets in all regions, for all subjects). We do not claim that that local activity and distal connectivity are completely independent, nor that they perfectly predict each other. Instead, we show that when considering distributed functional responses, connectivity is a powerful means of identifying voxels that afford discriminability of high-level object representations. Thus, the present findings do not contradict previous work but instead describe the relationship between connectivity and functional responses at a more complex level. Indeed, this is a valuable theoretical contribution given the widespread emphasis on distributed responses as a central functional organization principle of OTC (Haxby et al., 2001) and the wider brain.

Importantly, what exactly might account for the representational differences, at the level of multivoxel patterns, between most-connected and most-activated voxel sets? By definition, most-activated sets comprise voxels with the highest *t* values, potentially sampling from closely packed patches of voxels, whereas most-connected sets comprise a comparatively broader distribution of voxel responses. We speculate that these sets may differentially sample the heterogeneous functional responses of OTC. Although patchy organization of OTC is shown at a relatively coarse grain (e.g., OTC comprises sparsely distributed and largely nonoverlapping cortical patches that respond strongly to different types of information; Weiner and Grill-Spector, 2010), similar heterogeneous organization is also reflected at a finer spatial grain. For example, some voxel clusters within FFA respond preferentially to faces (compared with other objects), whereas other clusters show approximately equal tuning to multiple object categories (Grill-Spector et al., 2006; 2007; Hanson and Schmidt, 2011; Çukur et al., 2013); however, such responses may partially reflect responses to visual features that covary with certain categories rather than tuning to the categories themselves (Grill-Spector et al., 2006; Hanson and Schmidt, 2011; e.g., similar responses to faces and round-shaped objects, such as clocks or apples, for both voxels and single-cell recordings in macaque inferior temporal cortex; Tsao et al., 2006; Moeller et al., 2017). As such, distributed cortical representations are composed of heterogeneous voxel responses that reflect sensitivity to a diverse array of visual or semantic features, and such sparse encoding may allow for an exhaustive representational capacity of OTC via complex response patterns (Olshausen and Field, 2004; Grill-Spector et al., 2006).

Accordingly, we suggest that most-connected voxel sets may, in some cases, advantageously sample relatively more-diverse information than most-activated voxel sets. At a cognitive level, the connectivity-based voxel selection approach may better exploit computations occurring within heterogeneous patches dedicated to different types of domain-specific information. For instance, subsets of FFA voxels with strong connectivity to OFA may reflect greater tuning to face parts, whereas voxels that are well connected to STS may be preferentially tuned to dynamic-emotion-related information, potentially indexing the integration of information between different patches within a domain-specific network. Thus, voxel selection by connectivity recruits voxels that are highly connected with distal areas, bringing about a diverse set of object-related information. By contrast, selection by local activity targets voxels with strong amplitude responses that are potentially very important for particular computations at play within a given region. At the neural level, the connectivity-based approach may sample widely from functionally discrete patches, whereas the activity-based approach may draw more from spatially clustered sets of voxels with very similar (i.e., less informationally diverse) response profiles (Grill-Spector et al., 2006, 2007; Bell et al., 2009; Çukur et al., 2013).

For example, given five functionally discrete patches within a given target area, most-connected voxel sets may be more likely to sample from each patch than most-activated sets that may draw more heavily (and potentially, more redundantly) from fewer patches that exhibit strong, clustered amplitude responses. Thus, most-activated voxel sets may, in some cases, suffer from a higher degree of informational redundancy.

In the current study, we demonstrate higher decoding in most-connected than least-connected voxel sets when using *a priori* seed regions (e.g., tool-preferring PMTG, IPL, and SPL, were used as seeds for voxel selection and tool decoding in MFus), as motivated by highly correlated resting-state activity between areas that share category preferences (Zhang et al., 2009; Zhu et al., 2011; Stevens et al., 2015; Kamps et al., 2020). However, searchlight analyses revealed that regions outside these designated areas also afford similar effects, perhaps with some of these connections subserving both bottom-up and top-down modulations of local signal. These results are consistent with previous research showing that OTC subregions (e.g., FFA) show strong functional connectivity to regions that subserve more domain-general, and task-relevant, processing, that are often considered key nodes (e.g., posterior parietal cortex and inferior frontal gyrus) among attention- or cognitive-control networks (Cole et al., 2010; Vogel et al., 2012; Hutchinson et al., 2014).

We note that the central claim in this article—that local computations are influenced by connectivity to, and presumably via computations within, distal brain areas—is directionally agnostic; that is, from the present data, we cannot claim that local computations are causally influenced by connectivity to distal brain areas or vice versa. Instead, future research may address the causal nature of this relationship with neural disruption measures (e.g., transcranial magnetic stimulation) or brain lesion studies. We also note that the connectivity-based voxel selection approach used here is potentially generalizable to most other fMRI decoding experiments.

In conclusion, we show here that high-level multivariate representations in OTC can be reliably indexed by functional connectivity, demonstrating the importance of connectivity constraints on the complex functional organization of OTC.
