# Supplementary material for: High-Level Representations in Human Occipito-Temporal Cortex Are Indexed by Distal Connectivity
Source: J Neurosci. 2021 May 26;41(21):4678–85. doi: 10.1523/JNEUROSCI.2857-20.2021 (PMC8260247; doi:10.1523/JNEUROSCI.2857-20.2021)
Supplement: Figure 1-1 — Note: Three-way ANOVAs when comparing most-connected and least-connected voxel sets. Significant effects are indicated in bold; post hoc tests (following significant interactions involving the factor voxel selection are shown in gray cells. Download Figure 1-1, DOCX file. [file ns-JN-RM-2857-20-s01.docx]

*Figure 1-1*

| *Tools: 3-way repeated measures ANOVA* | |
| --- | --- |
| **Voxel selection** | **F(1,19) = 46.85, p < .001, η_p_^2^ = .711** |
| Region | F(1,19) = 3.84, p = .065, η_p_^2^ = .168 |
| **Decoding comparison** | **F(1,19) = 6.76, p = .018, η_p_^2^ = .262** |
| Voxel selection x region | F(1,19) = 0.00, p = .983, η_p_^2^ = .000 |
| Voxel selection x decoding comparison | F(1,19) = 0.47, p = .503, η_p_^2^ = .024 |
| Region x decoding comparison | F(1,19) = 1.18, p = .292, η_p_^2^ = .058 |
| Voxel selection x region x decoding comparison | F(1,19) = 1.36, p = .258, η_p_^2^ = .067 |
|  | |
| *Faces: 3-way repeated measures ANOVA* | |
| **Voxel selection** | **F(1,19) = 22.47, p < .001, η_p_^2^ = .542** |
| Region | F(1,19) = 1.03, p = .323, η_p_^2^ = .051 |
| **Decoding comparison** | **F(1,19) = 35.66, p < .001, η_p_^2^ = .652** |
| Voxel selection x region | F(1,19) = 1.21, p = .284, η_p_^2^ = .060 |
| **Voxel selection x decoding comparison** | **F(1,19) = 14.64, p < .001, η_p_^2^ = .435** |
| Region x decoding comparison | F(1,19) = 1.41, p = .249, η_p_^2^ = .069 |
| Voxel selection x region x decoding comparison | F(1,19) = 2.78, p = .112, η_p_^2^ = .128 |
| **MC > LC: Faces vs. Places** | **t(24.3) = 5.79, p < .001** |
| **MC > LC: Faces vs. Tools** | **t(24.3) = 3.09, p = .005** |
|  | |
| *Places: 3-way repeated measures ANOVA* | |
| **Voxel selection** | **F(1,19) = 47.03, p < .001, η_p_^2^ = .712** |
| **Region** | **F(1,19) = 14.99, p = .001, η_p_^2^ = .441** |
| **Decoding comparison** | **F(1,19) = 35.72, p < .001, η_p_^2^ = .653** |
| Voxel selection x region | F(1,19) = 0.52, p = .479, η_p_^2^ = .027 |
| **Voxel selection x decoding comparison** | **F(1,19) = 12.11, p = .003, η_p_^2^ = .389** |
| Region x decoding comparison | F(1,19) = 0.03, p = .869, η_p_^2^ = .001 |
| Voxel selection x region x decoding comparison | F(1,19) = 0.90, p = .354, η_p_^2^ = .045 |
| **MC > LC: Places vs. Faces** | **t(28.32) = 7.69, p < .001** |
| **MC > LC: Places vs. Tools** | **t(28.32) = 4.52, p < .001** |

Note: 3-way ANOVAs when comparing most-connected- and least-connected voxel sets. Significant effects are indicated in bold; post-hoc tests (following significant interactions involving the factor ‘voxel selection’) are shown in grey cells.
