# Supplementary material for: High-Level Representations in Human Occipito-Temporal Cortex Are Indexed by Distal Connectivity
Source: J Neurosci. 2021 May 26;41(21):4678–85. doi: 10.1523/JNEUROSCI.2857-20.2021 (PMC8260247; doi:10.1523/JNEUROSCI.2857-20.2021)
Supplement: Figure 1-2 — Note: Three-way ANOVAs when comparing most-connected and most-activated voxel sets. Significant effects are indicated in bold; post hoc tests (following significant interactions involving the factor voxel selection) are shown in gray cells. Download Figure 1-2, DOCX file. [file ns-JN-RM-2857-20-s02.docx]

*Figure 1-2*

| *Tools: 3-way repeated measures ANOVA* | |
| --- | --- |
| **Voxel selection (MC v MA)** | **F(1,19) = 14.96, p = .001, η_p_^2^ = .441** |
| **Region** | **F(1,19) = 11.76, p = .003, η_p_^2^ = .382** |
| Decoding comparison | F(1,19) = 4.22, p = .054, η_p_^2^ = .182 |
| **Voxel selection x region** | **F(1,19) = 10.84, p = .004, η_p_^2^ = .363** |
| Voxel selection x decoding comparison | F(1,19) = 0.81, p = .380, η_p_^2^ = .041 |
| Region x decoding comparison | F(1,19) = 0.84, p = .372, η_p_^2^ = .042 |
| **Voxel selection x region x decoding comparison** | **F(1,19) = 5.04, p = .037, η_p_^2^ = .210** |
| **MC > MA: MFus (TvF)** | **t(74.31) = 3.16, p = .002** |
| **MC > MA: MFus (TvP)** | **t(74.31) = 4.45, p < .001** |
| MC > MA: PMTG (TvF) | t(74.31) = 1.99, p = .050 |
| MC > MA: PMTG (TvP) | t(74.31) = -0.97, p = .335 |
|  | |
| *Faces: 3-way repeated measures ANOVA* | |
| **Voxel selection (MC v MA)** | **F(1,19) = 10.62, p = .004, η_p_^2^ = .358** |
| Region | F(1,19) = 2.39, p = .139, η_p_^2^ = .112 |
| **Decoding comparison** | **F(1,19) = 73.67, p < .001, η_p_^2^ = .795** |
| Voxel selection x region | F(1,19) = 0.26, p = .615, η_p_^2^ = .014 |
| Voxel selection x decoding comparison | F(1,19) = 0.16, p = .698, η_p_^2^ = .008 |
| Region x decoding comparison | F(1,19) = 0.87, p = .364, η_p_^2^ = .044 |
| Voxel selection x region x decoding comparison | F(1,19) = 1.94, p = .179, η_p_^2^ = .093 |
|  | |
| *Places: 3-way repeated measures ANOVA* | |
| Voxel selection (MC v MA) | F(1,19) = 0.03, p = .858, η_p_^2^ = .002 |
| Region | F(1,19) = 4.29, p = .052, η_p_^2^ = .184 |
| **Decoding comparison** | **F(1,19) = 42.66, p < .001, η_p_^2^ = .692** |
| Voxel selection x region | F(1,19) = 2.73, p = .115, η_p_^2^ = .126 |
| Voxel selection x decoding comparison | F(1,19) = 0.03, p = .862, η_p_^2^ = .002 |
| Region x decoding comparison | F(1,19) = 0.02, p = .900, η_p_^2^ = .001 |
| Voxel selection x region x decoding comparison | F(1,19) = 2.20, p = .154, η_p_^2^ = .104 |

Note: 3-way ANOVAs when comparing most-connected- and most-activated voxel sets. Significant effects are indicated in bold; post-hoc tests (following significant interactions involving the factor ‘voxel selection’) are shown in grey cells.
