# Supplementary material for: High-Level Representations in Human Occipito-Temporal Cortex Are Indexed by Distal Connectivity
Source: J Neurosci. 2021 May 26;41(21):4678–85. doi: 10.1523/JNEUROSCI.2857-20.2021 (PMC8260247; doi:10.1523/JNEUROSCI.2857-20.2021)
Supplement: Figure 2-1 — Note: Three-way ANOVAs when comparing most-connected and most-activated voxel sets (matched activation; p > .10). Significant effects are indicated in bold; post hoc tests (following significant interactions involving the factor voxel selection) are shown in gray cells. Download Figure 2-1, DOCX file. [file ns-JN-RM-2857-20-s03.docx]

*Figure 2-1*

| *Tools: 3-way repeated measures ANOVA (Matched activation: p>.10)* | |
| --- | --- |
| **Voxel selection** | **F(1,17) = 52.04, p < .001, η_p_^2^ = .754** |
| Region | F(1,17) = 3.29, p = .087, η_p_^2^ = .162 |
| **Decoding comparison** | **F(1,17) = 6.51, p = .021, η_p_^2^ = .277** |
| Voxel selection x region | F(1,17) = 0.20, p = .662, η_p_^2^ = .012 |
| Voxel selection x decoding comparison | F(1,17) = 0.50, p = .488, η_p_^2^ = .029 |
| Region x decoding comparison | F(1,17) = 2.15, p = .161, η_p_^2^ = .112 |
| Voxel selection x region x decoding comparison | F(1,17) = 2.36, p = .143, η_p_^2^ = .122 |
|  | |
| *Faces: 3-way repeated measures ANOVA (Matched activation: p>.10)* | |
| **Voxel selection** | **F(1,19) = 28.22, p < .001, η_p_^2^ = .598** |
| Region | F(1,19) = 0.67, p = .422, η_p_^2^ = .034 |
| **Decoding comparison** | **F(1,19) = 26.90, p < .001, η_p_^2^ = .586** |
| Voxel selection x region | F(1,19) = 0.47, p = .502, η_p_^2^ = .024 |
| **Voxel selection x decoding comparison** | **F(1,19) = 23.22, p < .001, η_p_^2^ = .550** |
| Region x decoding comparison | F(1,19) = 1.84, p = .190, η_p_^2^ = .088 |
| Voxel selection x region x decoding comparison | F(1,19) = 0.51, p = .485, η_p_^2^ = .026 |
| **MC > LC: Faces vs. places** | **t(23.57) = 6.60, p < .001** |
| **MC > LC: Faces vs. tools** | **t(23.57) = 3.43, p = .002** |
|  | |
| *Places: 3-way repeated measures ANOVA (Matched activation: p>.10)* | |
| **Voxel selection** | **F(1,17) = 50.92, p < .001, η_p_^2^ = .750** |
| **Region** | **F(1,17) = 12.04, p = .003, η_p_^2^ = .415** |
| **Decoding comparison** | **F(1,17) = 37.70, p < .001, η_p_^2^ = .689** |
| Voxel selection x region | F(1,17) = 2.12, p = .164, η_p_^2^ = .111 |
| **Voxel selection x decoding comparison** | **F(1,17) = 10.39, p = .005, η_p_^2^ = .379** |
| Region x decoding comparison | F(1,17) = 1.76, p = .202, η_p_^2^ = .094 |
| Voxel selection x region x decoding comparison | F(1,17) = 2.33, p = .145, η_p_^2^ = .121 |
| **MC > LC: Places vs. Faces** | **t(25.9) = 7.81, p < .001** |
| **MC > LC: Places vs. Tools** | **t(25.9) = 4.79, p < .001** |

Note: 3-way ANOVAs when comparing most-connected- and most-activated voxel sets (matched activation; p>.10). Significant effects are indicated in bold; post-hoc tests (following significant interactions involving the factor ‘voxel selection’) are shown in grey cells.
