# Supplementary material for: High-Level Representations in Human Occipito-Temporal Cortex Are Indexed by Distal Connectivity
Source: J Neurosci. 2021 May 26;41(21):4678–85. doi: 10.1523/JNEUROSCI.2857-20.2021 (PMC8260247; doi:10.1523/JNEUROSCI.2857-20.2021)
Supplement: Figure 2-2 — Note: Three-way and two-way ANOVAs when comparing most-connected and most-activated voxel sets (matched activation t 0 to −0.5). Significant effects are indicated in bold; post hoc tests (following significant interactions involving the factor voxel selection) are shown in gray cells. Download Figure 2-2, DOCX file. [file ns-JN-RM-2857-20-s04.docx]

*Figure 2-2*

| *Tools: 3-way repeated measures ANOVA (Matched activation: t 0 to -0.5)* | |
| --- | --- |
| **Voxel selection** | F(1,7) = 3.15, p = .119, η_p_^2^ = .310 |
| Region | F(1,7) = 0.78, p = .406, η_p_^2^ = .100 |
| **Decoding comparison** | F(1,7) = 0.06, p = .808, η_p_^2^ = .009 |
| Voxel selection x region | F(1,7) = 2.38, p = .167, η_p_^2^ = .254 |
| Voxel selection x decoding comparison | F(1,7) = 0.10, p = .763, η_p_^2^ = .014 |
| Region x decoding comparison | F(1,7) = 0.08, p = .785, η_p_^2^ = .011 |
| Voxel selection x region x decoding comparison | F(1,7) = 0.10, p = .757, η_p_^2^ = .015 |
|  | |
| *Tools (MFus): 2-way repeated measures ANOVA (Matched activation: t 0 to -0.5)* | |
| **Voxel selection** | **F(1,18) = 18.66, p < .001, η_p_^2^ = .509** |
| Decoding comparison | F(1,18) = 1.37, p = .258, η_p_^2^ = .071 |
| Voxel selection x decoding comparison | F(1,18) = 2.56, p = .127, η_p_^2^ = .125 |
|  | |
| *Tools (PMTG): 2-way repeated measures ANOVA (Matched activation: t 0 to -0.5)* | |
| Voxel selection | F(1,7) = 1.51, p = .258, η_p_^2^ = .178 |
| Decoding comparison | F(1,7) = 0.00, p = .993, η_p_^2^ = .000 |
| Voxel selection x decoding comparison | F(1,7) = 0.11, p = .746, η_p_^2^ = .016 |
|  | |
| *Faces: 3-way repeated measures ANOVA (Matched activation: t 0 to -0.5)* | |
| **Voxel selection** | **F(1,18) = 20.70, p < .001, η_p_^2^ = .535** |
| Region | F(1,18) = 0.83, p = .374, η_p_^2^ = .044 |
| **Decoding comparison** | **F(1,18) = 19.80, p < .001, η_p_^2^ = .524** |
| Voxel selection x region | F(1,18) = 0.14, p = .715, η_p_^2^ = .008 |
| **Voxel selection x decoding comparison** | **F(1,18) = 4.74, p = .043, η_p_^2^ = .208** |
| Region x decoding comparison | F(1,18) = 1.51, p = .234, η_p_^2^ = .078 |
| Voxel selection x region x decoding comparison | F(1,18) = 1.46, p = .242, η_p_^2^ = .075 |
| **MC > LC: Faces vs. places** | **t(24.97) = 5.04, p < .001** |
| **MC > LC: Faces vs. tools** | **t(24.97) = 3.26, p = .003** |
|  | |
| *Places: 3-way repeated measures ANOVA (Matched activation: t 0 to -0.5)* | |
| **Voxel selection** | **F(1,12) = 17.43, p = .001, η_p_^2^ = .592** |
| Region | F(1,12) = 3.52, p = .085, η_p_^2^ = .227 |
| Decoding comparison | F(1,12) = 0.57, p = .466, η_p_^2^ = .045 |
| Voxel selection x region | F(1,12) = 3.12, p = .103, η_p_^2^ = .207 |
| Voxel selection x decoding comparison | F(1,12) = 0.25, p = .625, η_p_^2^ = .021 |
| **Region x decoding comparison** | **F(1,12) = 5.07, p = .044, η_p_^2^ = .297** |
| Voxel selection x region x decoding comparison | F(1,12) = 0.76, p = .401, η_p_^2^ = .059 |
|  | |
| *Places (PPA): 2-way repeated measures ANOVA (Matched activation: t 0 to -0.5)* | |
| **Voxel selection** | **F(1,16) = 35.00, p < .001,** η_p_^2^ **= .686** |
| Decoding comparison | F(1,16) = 2.95, p = .105, η_p_^2^ = .156 |
| Voxel selection x decoding comparison | F(1,16) = 0.01, p = .939, η_p_^2^ = .000 |
|  | |
| *Places (OPA): 2-way repeated measures ANOVA (Matched activation: t 0 to -0.5)* | |
| Voxel selection | F(1,12) = 2.81, p = .120, η_p_^2^= .190 |
| Decoding comparison | F(1,12) = 2.23, p = .161, η_p_^2^ = .156 |
| Voxel selection x decoding comparison | F(1,12) = 0.01, p = .939, η_p_^2^ = .001 |

Note: 3-way- & 2-way ANOVAs when comparing most-connected- and most-activated voxel sets (matched activation t 0 to -0.5). Significant effects are indicated in bold; post-hoc tests (following significant interactions involving the factor ‘voxel selection’) are shown in grey cells.
